# Disruption of Erythritol Catabolism via the Deletion of Fructose-Bisphosphate Aldolase (Fba) and Transaldolase (Tal) as a Strategy to Improve the *Brucella* Rev1 Vaccine

**DOI:** 10.3390/ijms252011230

**Published:** 2024-10-18

**Authors:** Aitor Elizalde-Bielsa, Leticia Lázaro-Antón, María Jesús de Miguel, Pilar M. Muñoz, Raquel Conde-Álvarez, Amaia Zúñiga-Ripa

**Affiliations:** 1 Department of Microbiology and Parasitology, Instituto de Investigación Sanitaria de Navarra (IdiSNA), University of Navarra, 31008 Pamplona, Spain; aelizalde.7@alumni.unav.es (A.E.-B.); llazaro@ucdavis.edu (L.L.-A.); 2 Department of Medical Microbiology and Immunology, University of California, Davis, CA 95616, USA; 3 Department of Animal Science, Centro de Investigación y Tecnología Agroalimentaria de Aragón (CITA), 50059 Zaragoza, Spain; mjmiguel@cita-aragon.es (M.J.d.M.); pmmunnoz@cita-aragon.es (P.M.M.); 4 Instituto Agroalimentario de Aragón—IA2, CITA-Universidad de Zaragoza, 50009 Zaragoza, Spain

**Keywords:** *Brucella*, Rev1, vaccine, erythritol, Fba, Tal, trophoblasts, pregnancy safety, abortion, placenta

## Abstract

Brucellosis is a bacterial zoonosis caused by the genus *Brucella*, which mainly affects domestic animals. In these natural hosts, brucellae display a tropism towards the reproductive organs, such as the placenta, replicating in high numbers and leading to placentitis and abortion, an ability also exerted by the *B. melitensis* live-attenuated Rev1 strain, the only vaccine available for ovine brucellosis. It is broadly accepted that this tropism is mediated, at least in part, by the presence of certain preferred nutrients in the placenta, particularly erythritol, a polyol that is ultimately incorporated into the *Brucella* central carbon metabolism via two reactions dependent on transaldolase (Tal) or fructose-bisphosphate aldolase (Fba). In the light of these remarks, we propose that blocking the incorporation of erythritol into the central carbon metabolism of Rev1 by deleting the genes encoding Tal and Fba may impair the ability of the vaccine to proliferate massively in the placenta. Therefore, a Rev1Δ*fba*Δ*tal* double mutant was generated and confirmed to be unable to use erythritol. This mutant exhibited a reduced intracellular fitness both in BeWo trophoblasts and THP-1 macrophages. In the murine model, Rev1Δ*fba*Δ*tal* provided comparable protection to the Rev1 reference vaccine while inducing fewer adverse reproductive events in pregnant animals. Altogether, these results postulate the Rev1Δ*fba*Δ*tal* mutant as a reproductively safer Rev1-derived vaccine candidate to be studied in the natural host.

## 1. Introduction

Brucellosis is a widespread zoonosis caused by bacteria belonging to the genus *Brucella*, a group of facultative intracellular bacteria that includes several species with different host-preferences. Among them, the most relevant species, the so-called classical *Brucella* spp., infect domestic ruminants (*B. melitensis*, sheep and goats; *B. abortus*, cattle; and *B. ovis*, sheep), swine (*B. suis* biovars 1–3), and dogs (*B. canis*) [1,2,3].

In these natural hosts, the pathology features diverse reproductive symptoms related to the development of placentitis: reproductive failure with abortions/stillbirths and birth of weak offspring; infertility [4,5,6,7,8]; and reduced milk production due to infection and inflammation of mammary glands [7,9,10]. In this epizootiological context, *B. melitensis* is the most relevant species, as it is the most common cause of human infection, which can be acquired through direct contact with infected animals or consumption of unpasteurized milk products. This way, brucellosis not only represents a public health problem but also leads to important economic losses due to the reduction of livestock productivity, contributing to poverty in resource-limited regions [11,12]. Nonetheless, human-to-human transmission is anecdotal, and control of the disease relies on animal vaccination, for which the *B. melitensis* live-attenuated Rev1 strain is the only vaccine recommended by the World Organization for Animal Health for small ruminants [13,14]. Unfortunately, the Rev1 vaccine exerts several drawbacks, the most notable of which is its abortifacient effect when administered to pregnant animals. In endemic regions, such as parts of Africa and Asia, mass vaccination is the only viable strategy for controlling brucellosis [13]. However, in these areas, where sheep and goats breed all year round rather than seasonally (impairing the application of the vaccine in non-gestating periods), the use of Rev1 could lead to substantial economic losses due to abortion-related reproductive failures. There is therefore a clear need for improved reproductively safe *Brucella* vaccines that will help to control the disease in endemic areas.

The development of *Brucella* reproductive pathology is caused by the pronounced tropism of brucellae for the genital organs, among them the placenta, where brucellae replicate in exceedingly high numbers [15,16,17,18,19]. This active replication requires a very efficient use of the substrates available at the placental microenvironment. Among them, erythritol is thought to be one of the *Brucella*-preferred carbon sources during infection [20,21,22,23,24]. The *B. abortus* S19 vaccine, the other vaccine recommended by the World Organization for Animal Health but for cattle [13,14], is an attenuated strain that carries, among other modifications, a deletion in the erythritol catabolic operon affecting both *eryC* and *eryD* [25]. This deletion renders the S19 strain unable to use erythritol, an aspect that was initially related to the lower abortion induction of the vaccine [24,26]. However, further studies that restored both *eryC* and *eryD* showed that this did not restore the virulence of the strain [27]. This phenomenon may be explained by the other unknown genetic modifications of the S19 strain or the existence of a more complex metabolic scenario where erythritol is just one of several relevant substrates at the reproductive niche, as it is thought in the case of glycerol, lactate, and glutamate [28].

**Figure 1:**
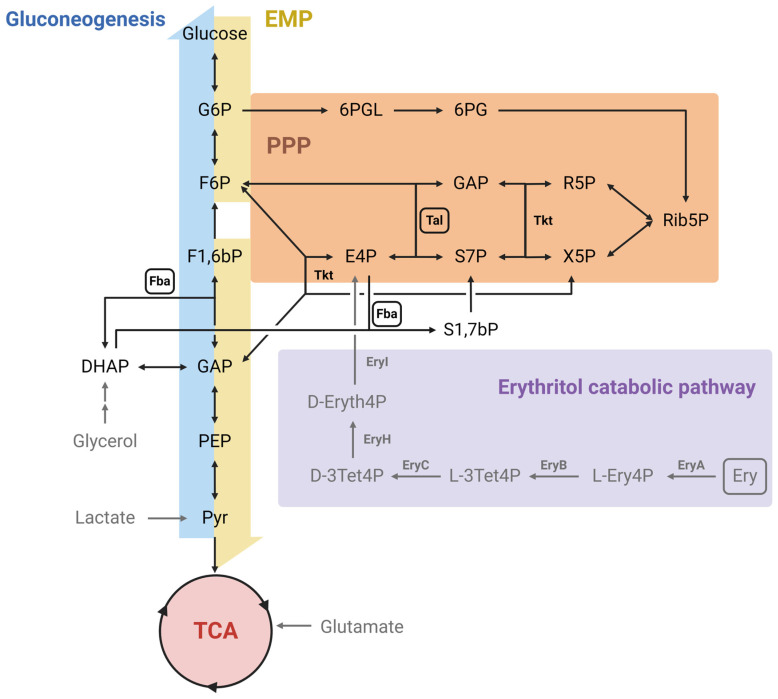
Central carbon metabolic network of *Brucella*. The metabolic network includes the gluconeogenic, Embden–Meyerhof–Parnas (EMP) and Pentose Phosphate (PPP) pathways, as well as the Tricarboxylic Acid cycle (TCA). Grey arrows and grey font indicate peripheral pathways. Metabolites: 6PG: 6-phosphogluconate; 6PGL: 6-P-gluconolactone; D-3Tet4P: D-3-Tetrulose-4-P; D-Eryth4P: D-Erythrulose-4-P; DHAP: dihydroxyacetone-P; E4P: erythrose-4-P; Ery: erythritol; F1,6bP: fructose-1,6-bisP; F6P: fructose-6-P; G6P: glucose-6-P; GAP: glyceraldehyde-3-P; L-3Tet4P: L-3-Tetrulose-4-P; L-Ery4P: L-Erythritol-4-P; PEP: phosphoenolpyruvate; Pyr: pyruvate; R5P: ribose-5-P; Rib5P: ribulose- 5-P; S1,7bP: sedoheptulose-1,7-bisP; S7P: sedoheptulose-7-P; X5P: xylulose-5-P. Enzymes: EryA: erythritol kinase; EryB: erythritol-1-P dehydrogenase; EryC: tetrulose-4-P racemase; EryH: D-3-tetrulose-4-P isomerase; EryI: D-erythrose-4-P isomerase; Fba: fructose bisP aldolase; Tal: transaldolase; Tkt: transketolase. Modified from [32].

In *Brucella*, erythritol is converted into erythrose-4-phosphate through a five-step pathway and introduced into the Pentose Phosphate pathway via the transaldolase- (Tal) and two transketolase (Tkt)-catalyzed reactions [29] (Figure 1). In this erythritol catabolic pathway, the first enzyme (the kinase EryA) carries an ATP-dependent phosphorylation that is futile when the downstream pathway is disrupted (i.e., mutations in *eryB*, *eryC*, *eryH*, or *eryI*), resulting in growth inhibition caused by ATP depletion [30]. In addition, in a recent publication, we described that the fructose-bisphosphate aldolase (Fba) exerts sedoheptulose-1,7-bisphosphate phosphatase activity in *B. suis* 513 (biovar 5), allowing a more efficient use of the abundant erythritol in the placenta and other reproductive organs [31].

Bearing the above in mind, we propose that blocking the incorporation of erythritol-derived erythrose-4-phosphate into the central carbon metabolism of Rev1 by deleting the genes encoding the transaldolase (Tal) and the fructose-bisphosphate aldolase (Fba) may impair the ability of the vaccine to proliferate massively in the placenta, thereby preventing the induction of abortion in pregnant ewes [31]. Therefore, in this work, we evaluated the impact of deleting *fba* and *tal* in the Rev1 vaccine background to develop a safer Rev1 vaccine.

## 2. Results and Discussion

### 2.1. Dysfunction of Fba and Tal Abrogates Rev1 Growth on Erythritol

The analysis of the Rev1 genome showed a high conservation of *fba* and *tal* (99.7% identity in both cases) when compared to *B. suis* 513, the strain where the Fba-bypass on erythritol catabolism was reported [31]. At the protein level, these sequence differences translated into minor amino acidic changes, namely [V156A] in Fba and [Y199D] in Tal between Rev1 and *B. suis* 513, that do not extend to the predicted functional regions of Fba or the described functional regions of Tal, according to the UniProt database [33]. Hence, we employed the genetic tools generated in our previous work [31] to construct the Rev1Δ*fba*, Rev1Δ*tal*, and Rev1Δ*fba*Δ*tal* mutants.

**Figure 2:**
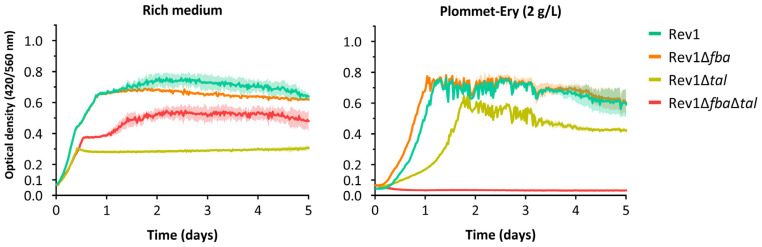
The double mutant Rev1∆*fba*∆*tal* is unable to grow on erythritol as the sole carbon source. Curve values at each time point represent the mean ± standard deviation (error bars are represented as the area within the respective values) of an experiment performed in technical triplicates. The experiment was repeated three times with similar results.

Growth studies of the mutant strains in rich medium showed no effect of single *fba* mutation on the growth of Rev1, consistent with previous findings in *B. suis* 513 [31], and an impaired bacterial growth in the case of Rev1Δ*tal* and Rev1Δ*fba*Δ*tal* (Figure 2). Intriguingly, the *tal* single mutant exhibited shorter generation times and lower growth yields than the double mutant Rev1Δ*fba*Δ*tal.* While the *tal* mutant phenotype is likely due to Rev1’s reliance on the Pentose Phosphate pathway as the only route for hexose metabolism [34], the reduced doubling time of the *fba*-*tal* mutant compared to Rev1Δ*tal* may be due to metabolic rearrangements caused by the absence of two key enzymes in the central carbon metabolism. In the case of growth on erythritol as a carbon source, only the double mutation on *fba* and *tal* completely ablated the growth of Rev1 (Figure 2), with the Rev1Δ*tal* growth curves showing a longer lag phase not detected for the Rev1Δ*fba* mutant, a phenomenon also observed for *B. suis* 513 [31]. These findings confirm the indispensability of both *fba* and *tal* for the growth of Rev1 on erythritol and support the existence of the newly described erythritol Fba-bypass [31] in other *Brucella* species, although further studies on other model *Brucella* spp. are required to reach more robust conclusions on the genus.

### 2.2. Rev1ΔfbaΔtal Is Attenuated in Trophoblastic and Macrophagic Cellular Models

Once we validated the incapability of Rev1Δ*fba*Δ*tal* to grow on erythritol, we continued with the characterization of this double mutant as a possible vaccine candidate. As a first approach to assess the residual virulence of the strain, we carried out cell infections in BeWo human trophoblasts and THP-1 human macrophage-like cells, representative cell types of brucellae-preferential host cells upon infection [8,35,36,37].

**Figure 3:**
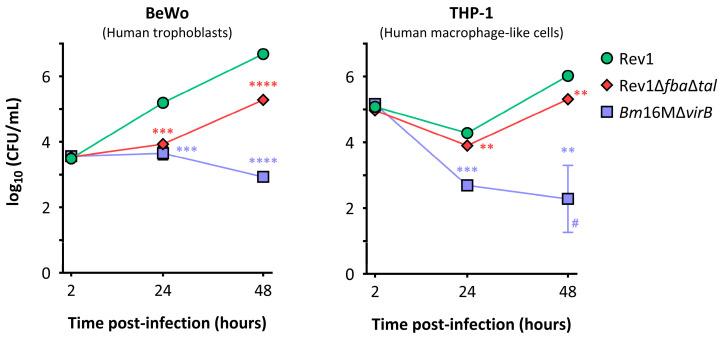
Rev1∆*fba*∆*tal* intracellular replication in BeWo human trophoblasts and THP-1 human macrophage-like cells. Bacterial replication levels were determined by CFU counting, and values are expressed as mean log_10_(CFU/mL) ± SD (# Some values fall under detection limit, DL = 1.22 log_10_[CFU/mL]); obtained from technical triplicates and performed three times, obtaining similar results. Statistical comparisons were made by unpaired *t*-test (** *p* < 0.01; *** *p* < 0.001; **** *p* < 0.0001).

In BeWo, Rev1Δ*fba*Δ*tal* showed virtually no increase in the number of intracellular CFUs during the first 24 h post-infection (p.i.) but a doubling time similar to that of Rev1 between the 24–48 h p.i. time-point, although reaching lower total CFU-values (Figure 3, left panel). Similar results were observed in THP-1, with Rev1Δ*fba*Δ*tal* showing a faintly increased intracellular killing compared to Rev1 at 24 h p.i. and slightly lower replication levels than the Rev1 during the 24–48 h p.i. period. (Figure 3, right panel).

These in vitro findings are in line with previous publications that found attenuation of erythritol catabolic mutants in other *Brucella* in human and murine cell lines. Barbier et al. showed that mutation of EryI, downstream of the first EryA kinase, resulted in attenuation of *B. abortus* 2308 in BeWo and THP-1 cells [38], probably due to the previously described EryA-mediated ATP depletion toxicity [30], a hypothesis that would also explain the attenuation profiles observed for *B. suis* 1330 *eryB*- and *eryC*-mutants in THP-1 cells or J774A.1 murine macrophages in studies prior to that of Barbier et al. [39,40]. Similarly, a *B. abortus* 2308 strain mutated in the erythritol operon promoter showed a mild attenuation in RAW264.7 mouse macrophages [41]. In this case, the results may not be as clear-cut since the deleted region could also contain the promoter of a series of genes encoded on the opposite strand. Additionally, the attenuation observed may be explained by its sensitivity to erythritol. Several years ago, erythritol was not detected in measurable quantities in the placentas of humans or mice [22]. However, Barbier et al. later found that, while this polyol is present, it is not essential for the multiplication of *B. abortus* in human and murine trophoblastic and macrophage-like cells or in the spleens and conceptuses of mice [38], which may account for a more complex metabolic scenario in which other preferred nutrients for *Brucella*, such as glycerol, lactate, or glutamate, also play a role in *Brucella* pathogenesis [28].

### 2.3. Rev1ΔfbaΔtal Protects at a Level Comparable to the Rev1 Vaccine in the Murine Model

**Figure 4:**
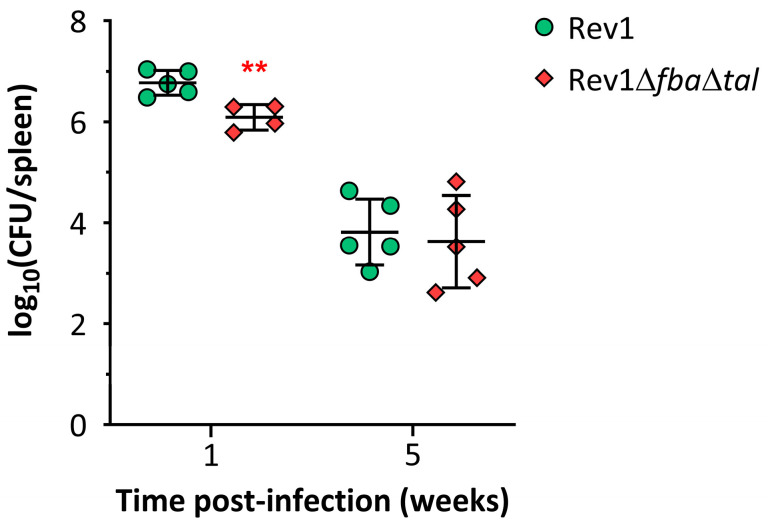
Rev1∆*fba*∆*tal* multiplication in the spleen of BALB/c mice. Mice (*n* = 5) were IP-infected with 10^5^ CFU/mouse of Rev1 or Rev1∆*fba*∆*tal* and euthanized at one and five weeks p.i. Bacterial burden at the spleens was determined by CFU counting and is expressed as mean log_10_(CFU/spleen) ± SD. Statistical comparisons at each time-point were made by unpaired *t*-test (** *p* < 0.01). The experiment was performed twice, obtaining similar results.

After the *in vitro* screening in cell cultures, we studied the attenuation of Rev1Δ*fba*Δ*tal* in the murine virulence model. For this purpose, we intraperitoneally infected BALB/c mice with 10^5^ CFU/mouse of Rev1Δ*fba*Δ*tal* or Rev1 and determined the splenic bacterial loads at one and five weeks p.i. (Figure 4). Rev1Δ*fba*Δ*tal* showed a slightly lower splenic bacterial recovery when compared to Rev1 at 1 week p.i., while the splenic CFU counts of the mutant at 5 weeks p.i. were similar to those of the Rev1 control (Figure 4).

The results regarding the effect of mutations in erythritol catabolic enzymes are diverse. While *B. suis* 1330 Δ*eryC* or *B. abortus* 2308 Δ*eryA* and Δ*eryH* mutants were attenuated at early days of infection in mice, only the *B. suis* mutant was attenuated at 56 days p.i. in BALB/C mice [40], with the *B. abortus* 2308 mutants showing no attenuation in C57BL/6 mice at 30 days p.i. [38]. On top of this, the study carried out by Zhang et al. found that mutation of the erythritol operon promoter greatly impacted the virulence in *B. abortus* 2308 in BALB/c mice in both acute and chronic phases of infection [41], although as mentioned, other possible effects due to the position of the deleted region need to be considered.

Nonetheless, despite this issue regarding solely the effect of erythritol use on virulence, it is still intriguing to find such an absence of attenuation for a mutant in enzymes also involved in key reactions of *Brucella* central carbon metabolism, suggesting potential compensating metabolic rearrangements able to restore bacterial fitness in vivo or the existence of a complex in vivo metabolic microenvironment in the spleen that renders the absence of these enzymes negligible. An example of these issues can be found in a recent work that describes an alternative pathway for the catabolism of erythritol in *B. abortus* 2308 via erythronate, an oxidative product of erythritol in the host [42]. In this work, the erythronate metabolic pathway was shown to cooperate with erythritol catabolism, playing an important role in pathogenesis. This erythritol derivate is catabolized to dihydroxyacetone-phosphate by *Brucella* and then fueled into the central carbon metabolism via Fba. In this regard, our Rev1Δ*fba*Δ*tal* vaccine candidate would exert an impact on the ability of *Brucella* to utilize erythritol through not only the classical erythritol catabolic pathway but also through the recently described *Brucella* erythronate pathway.

Once we evaluated the virulence of the Rev1Δ*fba*Δ*tal* vaccine candidate in the spleen of mice, we were encouraged to investigate its protective capacity. Hence, we vaccinated mice subcutaneously with 10^5^ CFU/mouse of our candidate or the Rev1 control and, four weeks after vaccination, challenged the mice with a virulent *B. melitensis* H38 strain. We observed that the Rev1Δ*fba*Δ*tal* vaccine candidate protected mice from *B. melitensis* H38 infection at the same level than the Rev1 vaccine and showed a reduced residual virulence when compared to Rev1 (Table 1).

Regarding protection, the study by Zhang et al. showed that BALB/c mice IP-vaccinated with 10^6^ CFU/mouse of the *B. abortus* 2308 erythritol promoter mutant exhibited a higher protective efficacy than the S19 vaccine control at two and four weeks post-challenge [41]. Although this was not the case in our study, our Rev1Δ*fba*Δ*tal* vaccine candidate protected mice against *B. melitensis* H38 infection, prompting the study of the reproductive safety of the vaccine candidate.

### 2.4. Rev1ΔfbaΔtal Is Reproductively Safer than the Rev1 Vaccine Strain in the Murine Model

Taking into account that *fba* and *tal* deletion did not affect Rev1 protection capacity and bearing in mind that the final goal of this work was to obtain a reproductively safe vaccine, we investigated the behavior of the Rev1Δ*fba*Δ*tal* mutant in pregnant mice. To this end, we evaluated the abortifacient effect of Rev1Δ*fba*Δ*tal* in pregnant mice, a model recently reviewed [43]. Briefly, we infected 8-day-pregnant CD-1 mice with 10^7^ CFU/mouse of Rev1Δ*fba*Δ*tal* or the Rev1 control vaccine and evaluated the pregnancy outcome.

In the mentioned review, we highlighted the need of a more informative reporting of adverse pregnancy outcomes in the pregnant mouse model of reproductive brucellosis, making emphasis not only on fetal viability but also on the effect of infection on other parameters, such as litter sizes [43]. In accordance, in this experiment, we found not only a differential effect on fetal viability but also on the litter sizes of mice infected with Rev1Δ*fba*Δ*tal* or the Rev1 vaccine control. To report this second effect on mouse pregnancy, we defined the “*Pregnancy Index*” as the proportion of fetuses that reach pregnancy term, whether viable or not, with respect to the PBS group (Table 2). Then, to integrate both effects on mouse pregnancy into a single gestational indicator, we also defined the “*Gestational Success Index*” as the proportion of fetuses that reach pregnancy term in a viable status with respect to the PBS group (i.e., the combined probability of the *Pregnancy Index* and the *Fetal Viability*) (Table 2), which may be a more complete indicator to describe the effects of *Brucella* infection on mouse pregnancy. Using the previously described parameters, we observed that the pups from the Rev1Δ*fba*Δ*tal* group showed an improved *Fetal Viability* when compared to the Rev1 control (89% vs. 6%, respectively; Table 2). Likewise, the dams infected with the mutant strain delivered larger litter sizes, resulting in an improved *Pregnancy Index* (72% vs. 57%, respectively; Table 2). This way, the Rev1Δ*fba*Δ*tal* effect on the pregnancy outcome led to an improved *Gestational Success Index* of 66%, while the Rev1-infected group yielded a *Gestational Success* of 6% (Table 2).

Taken together, the results obtained both in vitro and in vivo show that Rev1Δ*fba*Δ*tal* is a vaccine candidate with a comparable level of protection to that of the Rev1 reference vaccine but with a significantly decreased abortifacient effect, highlighting the role of placental erythritol in the development of placentitis and abortion. The approach of reducing erythritol catabolism has been recently proven effective with a Rev1 mutant strain carrying the same genomic deletion found in the S19 vaccine, displaying a reduced capacity to colonize and induce damage to the reproductive system of male goats [44]. As previously mentioned, the main novelty of this approach lies in the fact that the mutation of both *fba* and *tal* avoids alternative erythritol utilization pathways, such as the erythronate one [42]. Likewise, the Rev1Δ*fba*Δ*tal* would also carry an impaired ability to used gluconeogenic substrates [31], such as glycerol, lactate, or glutamate (Figure 1), the three of them also present preferentially in reproductive organs and proposed as *Brucella*-preferred carbon sources together with erythritol [28].

## 3. Conclusions

In summary, a Rev1Δ*fba*Δ*tal* double mutant was generated lacking the fructose-bisphosphate aldolase (Fba) and transaldolase (Tal) enzymes responsible of the final incorporation of erythritol through erythrose-4-P into the *Brucella* central carbon metabolism. Thus, Rev1Δ*fba*Δ*tal* was unable to use erythritol as the sole carbon source. The vaccine candidate showed a reduced intracellular fitness in BeWo trophoblasts and THP-1 macrophages when compared to the Rev1 control, while in the murine model, persisted and provided comparable protection to the Rev1 reference vaccine while inducing fewer adverse reproductive events in pregnant mice, giving the Rev1Δ*fba*Δ*tal* vaccine candidate significant improvements over the current Rev1 vaccine.

Altogether, this work provides a new basis for the development of safer *Brucella* vaccines by suppressing their ability to massively replicate in the placenta, which could also be applied to other *Brucella* spp. The final validation of Rev1Δ*fba*Δ*tal* in the natural host, in terms of residual virulence, protection, and reproductive safety, will help to verify this Rev1-derived candidate as a new improved vaccine against brucellosis with a lower abortifacient potential, which may facilitate the successful pursuing of mass vaccination campaigns in endemic areas.

## 4. Materials and Methods

### 4.1. Bacterial Strains and Plasmids

The bacterial strains and plasmids used in this work are listed in Tables S1 and S2 [45,46,47,48,49,50], respectively. All strains were stored at −80 °C in cryoprotector media: skim milk (Scharlau) or TYSB-7% DMSO (tryptic soy broth, Scharlau, supplemented with 0.5% yeast extract, Condalab, and dimethyl sulfoxide, VWR). All brucellae were handled under BSL-3 containment.

### 4.2. Culture Conditions

*Brucella* strains were routinely grown on solid or filtration-de-agarized BAB2 (Blood Agar Base No. 2; Oxoid, CM0271B) and *E. coli* strains on agar-supplemented TSB (i.e., TSA; Tryptic Soy Broth, Scharlau; European Bacteriological Agar, Condalab) at 37 °C. When indicated, the growth media were supplemented with 50 µg/mL kanamycin (Km; Sigma, Tokyo, Japan), 25 µg/mL nalidixic acid (Nal; Sigma), 1 mM 2,6-diaminopimelic acid (DAP, Sigma), 0.2% activated charcoal (Sigma), and/or 5% sucrose (PanReac AppliChem ITW Reagents).

The minimum medium used to study the in vitro phenotype of the *Brucella* metabolic mutants was the vitamin–salt mixture described by Plommet [51], modified as described by Barbier et al. [29]: 0.20 g/L thiamine HCl, 0.20 g/L nicotine acid, 0.07 g/L pantothenic acid, 0.10 g/L biotin, 0.50 g/L (NH_4_)_2_SO_4_, 9.20 g/L K_2_HPO_4_, 3 g/L KH_2_PO_4_, 0.10 g/L Na_2_HPO_4_, 10 g/L Mg_2_SO_4_, 0.11 mg/L Mn_2_SO_4_, 0.10 mg/L FeSO_4_, and 5 g/L NaCl and supplemented with 2 g/L of filtration-sterilized erythritol.

### 4.3. DNA Manipulations

Genomic sequences of *B. suis* 513 and *B. melitensis* Rev1 were obtained from the databases National Center for Biotechnology Information (NCBI) and Kyoto Encyclopedia of Genes and Genomes (KEGG). Searches for DNA and protein homologies were carried out using NCBI BLAST [52]. Sequence alignments were performed with Clustal Omega [53,54]. Primers were synthesized by Sigma (Haverhill, UK) or Condalab (Madrid, Spain). DNA sequencing was performed by Secugen (Madrid, Spain). Plasmid and chromosomal DNA were extracted with QIAprep^®^ Spin Miniprep and QIAamp^®^ DNA Mini Kit (Qiagen, Hilden, Germany), respectively.

### 4.4. Mutagenesis

Rev1∆*fba* deleted in *fba* (*B. melitensis* Rev1 homolog, genome not annotated, of *B. abortus* BAB2_0365) was obtained by double recombination, introducing the suicide plasmid pAZI-38 [31] (Table S2) into Rev1 by conjugation [55,56]. Exconjugants that integrated the plasmid were selected with Km. Then, the loss of the plasmid causing either a deletion or a sibling revertant wild type was selected on 5% sucrose. The resulting clones were screened by PCR with the primers Fba-F1 (5′-GCGGCCTGTTTTTCTATGTG-3′) and Fba-R4 (5′-CGGAAGTGGCAAAGACCAT-3′), which amplified a fragment of 559 bp in the mutants and 1513 bp in the sibling revertants. The absence/presence of the deleted sequence in these clones was verified using the primer Fba-R5 (5′-GCTCACCTTCCACCGAAAT-3′) hybridizing in the deleted region.

To construct the mutants Rev1∆*tal* and Rev1∆*fba*∆*tal* deleted in *tal* (*B. melitensis* Rev1 homolog, genome not annotated, of *B. abortus* BAB1_1813), the mutator plasmid pLLA-18 [31] (Table S2) was introduced in Rev1 by conjugation, as previously described [56]. Exconjugants that integrated the plasmid were selected with Km. After allelic exchange, the loss of the plasmid was selected, as described above, on 5% sucrose. The resulting clones were screened by PCR with the primers Tal-F1 (5′-CGGGCAATTGAAAACTTCTG-3′) and Tal-R4 (5′-CAGGTTCGCAAATTCCTGAC-3′), which amplified a fragment of 707 bp in the mutants and 1280 bp in the sibling revertant clones. The absence/presence of the deleted sequence in these clones was verified using the primer Tal-R5 (5′-AAATCTACCCGCGCTCATTA-3′) hybridizing in the deleted region.

### 4.5. Growth Curves

For the growth curve studies, bacteria were grown in BAB2 broth at 37 °C with orbital agitation. After 18 h of incubation, bacteria were harvested by centrifugation at 15,700× *g* for 5 min and resuspended in 10 mL of Plommet’s medium at an optical density at 600 nm (OD600) of 0.1. After another 18 h of incubation with agitation at 37 °C, bacteria were harvested again by centrifugation and resuspended at an OD600 = 0.1 in 1 mL of the medium. The bacterial inocula were then transferred to the Bioscreen plates (200 μL/well), and growth was monitored as the absorbance at 420–580 nm in a Bioscreen C (Lab Systems) every 30 min, with continuous shaking at 37 °C. Growth curve studies were repeated three times.

### 4.6. Cell Line Infections

BeWo human trophoblasts (ATCC® CCL-98™ [57]) were routinely cultured in F-12K medium (Kaighn’s Modification of Ham’s F-12 Medium; ATCC^®^) supplemented with 10% FBS (Fetal Bovine Serum; Sigma) and THP-1 macrophage-like cells (ATCC® TIB-202™ [58]) in RPMI-1640 (Roswell Park Memorial Institute 1640 Medium; Gibco, MA, USA) supplemented with 10% FBS and 100 U/mL penicillin and 100 µg/mL streptomycin (Gibco). Both cell lines were maintained at 37 °C with a 5% CO_2_ atmosphere for at least one week prior to infection and tested negative for *Mycoplasma* prior to each experimental assay employing the LooKOut^®^ *Mycoplasma* PCR Detection Kit (Sigma).

Infections were performed as described elsewhere [38,59]. BeWo cells were seeded one day prior infection in 24-well plates at 2 × 10^4^ cells/well, and THP-1 cells were seeded at 1 × 10^5^ cells/well 48 h prior to infection, followed by monocyte-to-macrophage differentiation with 50 ng/mL phorbol 12-myristate 13-acetate (Abcam, ab120297) at 24 h pre-infection. On infection day, cells were counted and infected with a multiplicity of infection (MOI) of 100:1. After a centrifugation step at 400× *g* for 10 min at 4 °C, cells were incubated for 30 min at 37 °C with 5% CO_2_. Then, to remove extracellular bacteria, cells were washed with fresh medium and incubated for 1 h with complete medium supplemented with 100 µg/mL of gentamicin. After that, the cells were maintained with medium containing 25 µg/mL of gentamicin. Throughout the infection assays, the cells were monitored daily on a light-inverted microscope, and no remarkable infection-related morphological changes were observed. At 2, 24, and 48 h p.i., cells were lysed with 0.1% Triton X100 in DPBS (Dulbecco’s Phosphate Buffered Saline; Gibco) for 5 min at room temperature. After the detergent treatment, the lysates were collected, 10-fold diluted, and plated on BAB2 to determine the number of intracellular bacteria. All experiments were performed in triplicate, and the Rev1 strain was used as a parental control and a *virB10* mutant [60] as an attenuation control. Results are expressed as mean log_10_(CFU/mL) ± SD (*n* = 3; repeated three times with similar results), and statistical comparisons with respect to the Rev1 control were made with GraphPad (version 9) using unpaired *t*-test.

### 4.7. Mouse Model Assays

#### 4.7.1. Virulence and Protection Model

Seven-week-old female BALB/c mice (Harlan Laboratories, Bicester, UK) were housed in the BSL3 facilities of CITA; (ES502970012025 and A/ES/17/I-30) for 1 week before and during the experiments, with water and food ad libitum. The animal handling and other procedures were in accordance with the current European (directive 2010/63/UE) and Spanish (RD 53/2013) legislations, supervised by the Ethical Committee for Animal Experimentation and authorized by Aragón Government (reports No. 2020-03 and 2020-04).

For the virulence studies, groups of ten BALB/c mice were inoculated intraperitoneally with 10^5^ CFU/mouse of the corresponding strain in 0.1 mL of buffered saline solution (BSS; 0.015 M NaCl, 7 mM KH_2_PO_4_, and 10 mM K_2_HPO_4_; pH 6.85), and doses were retrospectively assessed by plating inocula countable dilutions. The Rev1 strain was used as a parental control. Animal welfare was tracked daily, and no signs of illness due to inoculation with *Brucella* were found. At one and five weeks post-infection, five mice per group were euthanized by cervical dislocation, and the mean CFU values per spleen were determined as reviewed elsewhere [61]. The identity of the infecting strain was confirmed by PCR from the isolates obtained from each individual mouse. Results are expressed as mean log_10_(CFU/spleen) ± SD (*n* = 5), and statistical comparisons between the vaccine candidates and the Rev1 control were made using unpaired *t*-test with GraphPad (version 9).

For the protection studies, groups of four BALB/c mice were inoculated subcutaneously with 10^5^ CFU/mouse of the Rev1∆*fba*∆*tal* mutant. The Rev1 reference vaccine (10^5^ CFU/mouse) or mice inoculated with BSS were used as effective-vaccine and unvaccinated controls, respectively. Four weeks after vaccination, all animals were challenged intraperitoneally with 10^4^ CFU of the virulent H38::Tn7KmR strain (Aragón-Aranda et al., unpublished results; Table S1). As mentioned above, the animals were tracked daily to ensure their welfare and check on any potential health issue. After 2 weeks, CFU/spleen numbers of the challenge strain were determined by plating on BAB2 supplemented with Km. Values of residual vaccine were also calculated by subtracting CFU numbers on Km from those obtained on BAB2. The identity of the strain isolates from each individual mouse was confirmed by PCR. Results are expressed as mean log_10_(CFU/spleen) ± SD (*n* = 5), and statistical comparisons between vaccines and controls for challenge strain values were made with GraphPad (version 9) using ANOVA and Fisher’s Protected Least Significant Differences (PLSD) tests.

#### 4.7.2. Pregnancy Safety Model

Six-to-eight-week-old SWISS (RjOrl:SWISS [CD-1^®^]; henceforth CD-1) pregnant mice were purchased from JanvierLabs (Le Genest-Saint-Isle, France). Animals were allocated in microisolator cages with water and food ad libitum at the Department of Microbiology and Parasitology BSL3 facilities (A/ES/18/I-22) at arrival and during infection. Animal handling and procedures were in accordance with the current European (directive 86/609/EEC) and Spanish (RD 53/2013) legislations, supervised by the corresponding Ethical and Animal Welfare Committee of the Institution and authorized by Gobierno de Navarra (Protocol number CEEA-R076-20).

Groups of seven CD-1 pregnant mice were intraperitoneally infected at day 8 postconception with approximately 10^7^ CFU of Rev1∆*fba*∆*tal* in 0.1 mL of PBS, and doses were retrospectively assessed by plating inocula countable dilutions. The Rev1 strain or mice inoculated with PBS were used as controls of the normal abortifacient effect of the reference vaccine and the normal pregnancies in our mouse model, respectively. Animal welfare was tracked daily, and no signs of illness due to inoculation with *Brucella* were found. On day 18 post-conception, mice were euthanized by cervical dislocation, and the gestating uterus was isolated and further dissected. The pregnant status of the dam and the number of pups/dam were noted and, also, the alive/death status of the fetuses was determined by the observation of vitality and movement immediately after uterus exposition and further dissection, as well as by body development and size and skin color. Accordingly, the corresponding pregnancy indexes were calculated: *Fetal Viability*, the proportion of viable fetuses at term with respect to the litter size in each mouse; *Pregnancy Index*, the proportion of fetuses that reach pregnancy term, whether viable or not, with respect to the PBS group; and *Gestational Success Index*, the proportion of fetuses that reach pregnancy term in a viable status with respect to the PBS group, i.e., the mathematical product of the *Pregnancy Index* and *Fetal Viability*.

## Figures and Tables

**Table 1:** Protection induced by Rev1Δ*fba*Δ*tal* against *B. melitensis* H38. Mice (*n* = 5) were subcutaneously vaccinated with 10^5^ CFU/mouse of Rev1 or Rev1Δ*fba*Δ*tal* and challenged 4 weeks later with *B. melitensis* H38; 2 weeks later, mice were euthanized and protection units determined as the reduction in splenic bacterial burden of the challenge strain.

	Mean log_10_ (CFU_H38_/Spleen) ± SD		
Vaccine	*B. melitensis* H38	Vaccine	Protection Units
Rev1Δ*fba*Δ*tal*	2.21 ± 2.21 ^a^	1.21 ± 1.10 ^b^	3.69 ^b^
Rev1	1.05 ± 1.54 ^a^	2.18 ± 1.34	4.85
Unvaccinated	5.89 ± 0.27	-	-

Statistical comparison of mean log_10_ *B. melitensis* H38 CFU/spleen: ^a^ *p* < 0.01 vs. unvaccinated. ^b^ No significant difference was found between Rev1 and Rev1Δ*fba*Δ*tal* in either protection or residual virulence.

**Table 2:** Reproductive safety of Rev1Δ*fba*Δ*tal***.** Mice (*n* = 7) were intraperitoneally infected with 10^7^ CFU/mouse of Rev1 or Rev1Δ*fba*Δ*tal* on day 8 post-conception and sacrificed at day 18 post-conception.

	Gestational Indicators (%) ^a^		
Strain	*Fetal Viability* ^b^	*Pregnancy Index* ^c^	*Gestational Success Index* ^d^
Rev1Δ*fba*Δ*tal*	89 ± 27	72 ± 28	66 ± 36
Rev1	6 ± 14	57 ± 24	6 ± 14
PBS	100 ± 0	-	-

^a^ Data shown in the table corresponds to the averaged individual gestational indicator values obtained for each mouse in the corresponding experimental group. ^b^ *Fetal Viability*: Proportion of viable fetuses at term with respect to the litter size in each mouse. ^c^ *Pregnancy Index*: Proportion of fetuses that reach pregnancy term, whether viable or not, with respect to the PBS group. ^d^ *Gestational Success Index*: Proportion of fetuses that reach pregnancy term in a viable status with respect to the PBS group, i.e., *Pregnancy Index · Fetal Viability*.

## Data Availability

The raw data supporting the conclusions of this article will be made available by the authors on request.

## Supplementary Materials

The following supporting information can be downloaded at https://www.mdpi.com/article/10.3390/ijms252011230/s1.
