# Supplementary material for: Disruption of Erythritol Catabolism via the Deletion of Fructose-Bisphosphate Aldolase (Fba) and Transaldolase (Tal) as a Strategy to Improve the Brucella Rev1 Vaccine
Source: Int J Mol Sci. 2024 Oct 18;25(20):11230. doi: 10.3390/ijms252011230 (PMC11508834; doi:10.3390/ijms252011230)
Supplement: Supplementary file 1 [file ijms-25-11230-s001.zip › ijms-3209018-supplementary.pdf]

Table S1. Bacterial strains constructed or used in this work.

Strains	Characteristics [1]	Source or reference
Brucella		
***B. melitensis* Rev1**	*B. melitensis* Rev1 vaccine reference strain.	CITA collection
Rev1Δ*fba*	Rev1 deleted in *fba* (fructose-bisphosphate aldolase) using pAZI-38 (Lázaro-Antón, 2019).	This work
Rev1Δ*tal*	Rev1 deleted in *tal* (transaldolase) using pLLA-18 (Lázaro-Antón, 2019).	This work
Rev1Δ*fba*Δ*tal*	Rev1 deleted in *fba* (fructose-bisphosphate aldolase) using pAZI-38 (Lázaro-Antón, 2019) and in *tal* (transaldolase) using pLLA-18 (Lázaro-Antón, 2019).	This work
***B. melitensis* 16M**	*B. melitensis* biovar 1 16M strain, Nal^R spontaneous mutant; virulent.	(González et al., 2008)
*Bm*16MΔ*virB*	*B. melitensis* 16M deleted in *virB10* (type IV secretion system).	CIML collection
***B. melitensis* H38**	*B. melitensis* biovar 1 virulent *strain*	(González et al., 2008)
B. melitensis H38::Tn7Km^R	*B. melitensis* biovar 1 H38 strain carrying a miniTn7 transposon (pUC18R6KT-mini-Tn7T-Km); Km^R; challenge strain.	Aragón-Aranda et al. unpublished results
E. coli		
**DH5α**	F- Φ80lacZΔM15 Δ(lacZYA-argF) U169 recA1 end A1 hsdR17(rk-, mk+) phoA supE44 thi-1 gyrA96 relA1 λ-	Invitrogen
**β2150**	*thrB1004 pro thi strA hsdS lacZΔM15* (F′*lacZΔM15 lacI^q^ proA^+^ proB^+^*) Δ*dapA::erm* (Erm^r) *pir*. *E. coli* deficient in the DAP (2,6-diaminopimelic acid) synthesis	(Dehio & Meyer, 1997)
**S17λpir**	Tp^R $Strp^R$ *recA thi hsdRM*+, lambda pyr phage lysogen RP4::2-Tc::Mu::Km Tn7	(Miller & Mekalanos, 1988; Simon et al., 1983)

[1] Abbreviations: Amp, ampicillin; DAP, 2,6-diaminopimelic acid; Km, kanamycin; Nal, nalidixic acid; ^{R}, resistant; Strp, streptomycin; Tc, tetracycline; Tp, trimethoprim.

Table S2. Vectors or plasmids used in this work.

Vectors / Plasmids	Characteristics [1]	Source or reference
pJQKm	Suicide vector; Km^R; Sac^S	(Scupham & Triplett, 1997)
pRK2013	Helper vector containing *tra* and *mob* genes	(Figurski & Helinski, 1979)
pAZI-38	pJQKm suicide plasmid containing the *fba* deleted allele	(Lázaro-Antón et al., 2024)
pLLA-18	pJQKm suicide plasmid containing the *tal* deleted allele	(Lázaro-Antón et al., 2024)

[1] Abbreviations: Km, kanamycin; ^{R}, resistant; Sac, sucrose; ^{S}, sensitive.
